# PI3K/AKT/mTOR Signaling Pathway Is Required for JCPyV Infection in Primary Astrocytes

**DOI:** 10.3390/cells10113218

**Published:** 2021-11-18

**Authors:** Michael P. Wilczek, Francesca J. Armstrong, Colleen L. Mayberry, Benjamin L. King, Melissa S. Maginnis

**Affiliations:** 1 Department of Molecular and Biomedical Sciences, University of Maine, Orono, ME 04469, USA; michael.wilczek@maine.edu (M.P.W.); francesca.armstrong@maine.edu (F.J.A.); colleen.mayberry@maine.edu (C.L.M.); benjamin.l.king@maine.edu (B.L.K.); 2 Graduate School in Biomedical Sciences and Engineering, University of Maine, Orono, ME 04469, USA

**Keywords:** JC polyomavirus, PML, astrocytes, SVGA cells, primary cells, PI3K, AKT, mTOR, rapamycin, wortmannin

## Abstract

Astrocytes are a main target of JC polyomavirus (JCPyV) in the central nervous system (CNS), where the destruction of these cells, along with oligodendrocytes, leads to the fatal disease progressive multifocal leukoencephalopathy (PML). There is no cure currently available for PML, so it is essential to discover antivirals for this aggressive disease. Additionally, the lack of a tractable in vivo models for studying JCPyV infection makes primary cells an accurate alternative for elucidating mechanisms of viral infection in the CNS. This research to better understand the signaling pathways activated in response to JCPyV infection reveals and establishes the importance of the PI3K/AKT/mTOR signaling pathway in JCPyV infection in primary human astrocytes compared to transformed cell lines. Using RNA sequencing and chemical inhibitors to target PI3K, AKT, and mTOR, we have demonstrated the importance of this signaling pathway in JCPyV infection of primary astrocytes not observed in transformed cells. Collectively, these findings illuminate the potential for repurposing drugs that are involved with inhibition of the PI3K/AKT/mTOR signaling pathway and cancer treatment as potential therapeutics for PML, caused by this neuroinvasive virus.

## 1. Introduction

JC polyomavirus (JCPyV) is a human-specific pathogen and is the causative agent of a fatal disease in the central nervous system (CNS) known as progressive multifocal leukoencephalopathy (PML) [1,2,3,4]. The virus is present in ~60 to 80% of the adult population, where initial infection is thought to occur in tonsillar tissue [5,6,7,8], allowing for secondary infections in circulating B cells, bone marrow, and kidneys [7]. In nearly all infected individuals, JCPyV is characterized as a persistent, asymptomatic infection, and the virus is periodically shed in the urine [5,6,7,9,10,11]. However, during immunosuppression, JCPyV can reactivate and spread to the CNS [9,12,13], causing the fatal, demyelinating disease PML [1,2,3,4]. Due to the immunosuppressive state associated with HIV infection, a large proportion of individuals diagnosed with PML are infected with HIV; however, due to more effective treatments related to HIV/AIDS, PML incidence is decreasing in this population [14,15,16]. Unfortunately, new risk groups are emerging, with a higher proportion of PML cases diagnosed in patients with hematological malignancies and in patients taking immunomodulatory therapies for immune-mediated diseases [16]. Specifically, individuals with multiple sclerosis (MS) undergoing natalizumab treatment are significantly at risk for PML development [16,17,18,19]. Current treatment for PML is focused on removal of immunosuppressive therapies or treatment of the underlying immunosuppression [19,20,21,22]. Additional treatment options, including adoptive T cell transfer and checkpoint inhibitors, prolong life expectancy; however, these treatments are still relatively new, only address the underlying immunosuppression and can result in severe morbidity [19,20,23,24,25]. 

In the CNS, astrocytes are a main target during JCPyV infection [26]. The lytic destruction of these cells, along with JCPyV infection of oligodendrocytes, leads to PML [1,2,3,4]. Once a cell becomes infected, JCPyV hijacks the host cell machinery to produce viral proteins, including Large T Antigen (T Ag), to create a favorable environment for JCPyV DNA replication; this occurs through the binding of T Ag to retinoblastoma (Rb) and sequestering p53 [27,28]. As transformation ensues and viral DNA replication progresses, the late gene expression of JCPyV occurs [28,29]. Here, viral proteins (VP) 1, 2, and 3 are transcribed and translated to encapsidate the viral DNA [30], before subsequent release of the newly formed virions into the surrounding environment. 

Initial studies were performed to better understand JCPyV infection in astrocytes, and the infectious cycle was compared to infection in SVG-A cells (SVGAs) [31]. SVGAs are a mixed population of glial cells, mostly comprised of astrocytes, immortalized with SV40 T Ag to support robust levels of JCPyV infection [32,33,34]. Our previous research demonstrated that the infectious cycle was delayed in primary normal human astrocytes (NHAs) [31]. This delay was most likely the consequence of SV40 T Ag, as the immortalization of NHAs with this protein (referred to as NHA-Ts) resulted in levels of infection comparable to that observed in SVGAs [31]. In addition, differences in JCPyV infection between cell types was attributed to variation in cyclin expression [31]. Cyclins are commonly used as markers for the cell cycle [26,35,36,37], and it was demonstrated that JCPyV infection was able to drive the cell into S phase by the accumulation of cyclin E in the nucleus of NHAs [31]. The S phase is needed for successful DNA viral replication [28]. However, as the infectious cycle ensued, cyclin B1, a marker of the G_2_/M phase, accumulated in the cytoplasm of NHAs, allowing for productive viral infection; this accumulation of cyclin B1 was not observed during infection of immortalized cells at the same time points [31].

SV40 T Ag is known to dysregulate the cell cycle and activate cellular pathways that can potentially confound mechanisms during JCPyV infection [28,38]. One of these pathways is the AKT signaling pathway, important in cell growth and survival [39,40,41,42,43,44,45]. Previous research has demonstrated that SV40 T Ag can inhibit apoptosis in *ts*13 cells by activating the AKT signaling pathway and can directly phosphorylate AKT in U2OS cells [46,47]. In relation to this, our understanding of the AKT signaling pathway during JCPyV infection is limited. Currently, it has been shown that a component of the AKT pathway, specifically phosphoinositide 3-kinase γ (PI3Kγ), is involved in JCPyV infection in cells immortalized with SV40 T Ag, such as SVGAs; the authors hypothesize that this pathway was activated upon stimulation of G protein-coupled receptors (GPCRs) [48]. However, PI3K signaling is complex and is activated by various mechanisms, including Ras, a component of the mitogen-activated protein kinase (MAPK) pathway, and has numerous isoforms defined by their catalytic and regulatory subunits [49]. As a result of SV40 T Ag activating AKT, and being able to influence other pathways, it is unclear if the previously identified PI3K/AKT signaling pathway was due to the immortalization of cells by SV40 T Ag or by JCPyV infection. Additionally, genes downstream of AKT, such as mechanistic target of rapamycin (mTOR), have also been implicated in JCPyV infection [50]. Overall, these experiments demonstrated the importance of PI3Kγ and mTOR [48,50], but due to the consequences of immortalization, additional research should elucidate these proteins in a primary cell line.

There have been many advances in understanding the PI3K/AKT/mTOR signaling pathway, as this pathway is frequently altered in human cancer [51]. As a result, there have been numerous chemical inhibitors developed targeting the PI3K/AKT/mTOR signaling pathway, some of which are in clinical trials or are already FDA approved [52]. This includes MK2206, an AKT inhibitor [53], and rapamycin, also known clinically as sirolimus (the generic drug name), an mTOR inhibitor, that has been FDA-approved for decades and used to prevent organ transplant rejection and to protect coronary stents [54,55,56]. Additionally, a clinical study has demonstrated that mTOR inhibitors, such as rapamycin, can cross the blood–brain barrier (BBB), providing a premise for the treatment of neurological disorders [57,58]. 

Along with the diverse drugs targeting this pathway and their ability to traverse the BBB, they have also been implicated in JCPyV infection, contributing to a framework for future PML therapeutics. MK2206 has been demonstrated to reduce JCPyV DNA replication in an oligodendrocyte cell line [59]; however, mTOR inhibitors, such as rapamycin, increased expression of JCPyV T Ag in an immortalized kidney cell line—human embryonic kidney (HEK) 293 cells [50]. Due to these recent findings, the lack of an effective treatment for PML, and observed differences in JCPyV infection in immortalized cells, components of the PI3K/AKT/mTOR signaling pathway were analyzed through RNA sequencing (RNA-seq) analysis, and chemical inhibitors that target this signaling pathway were examined for their capacity to reduce JCPyV infection. These studies were conducted in primary human astrocytes (i.e., NHAs) and compared to cell types immortalized with SV40 T Ag, SVGAs, and NHA-Ts, to determine whether transformation of cells with T Ag yielded differing outcomes in activation of the PI3K/AKT/mTOR pathway and the JCPyV infectious cycle. This study also further characterizes the mechanisms of JCPyV infection of NHAs and describes how additional cellular pathways are possibly intertwined, such as pathways that are required to transform the cell, to support viral infection.

## 2. Materials and Methods

### 2.1. Cells and Viruses

The maintenance of primary normal human astrocytes (i.e., NHAs) has been previously described [31]. In brief, NHAs were purchased from Lonza Walkersville Inc. (Walkersville, MD, USA), where they were isolated from a 19-week-gestation female with no detected levels of HIV, hepatitis B virus (HBV), or hepatitis C virus (HCV). They were cultured according to the manufacturer’s guidelines in astrocyte growth medium and supplemented with SingleQuots supplements (Lonza Walkersville Inc. (Walkersville, MD, USA)) and 1% penicillin-streptomycin (P-S) (Corning, Corning, NY, USA). All experiments were performed at low passages (P2 to P10). SVGAs were graciously provided by the Atwood Laboratory (Brown University). They were cultured in complete minimum essential medium (MEM) (Corning, Corning, NY, USA), with 10% fetal bovine serum (FBS) (Atlanta Biologicals, Bio-Techne, Flowery Branch, GA, USA), 1% P-S, and 0.1% Plasmocin prophylactic (InvivoGen, San Diego, CA, USA). All cell types were grown in a humidified incubator at 37 °C with 5% CO_2_. The generation of NHA-Ts has been previously described [31]; they were cultured similarly to SVGAs, however, 16% FBS was used. The expression of SV40 T Ag in NHA-Ts was consistently monitored using epifluorescence microscopy staining for SV40 T Ag (Table 1 below). All cell types were grown in a humidified incubator at 37 °C with 5% CO_2_. JCPyV strains were provided by the Atwood Laboratory (Brown University, Providence, RI, USA). The generation and production of the lysate viral strains of Mad-1/SVEΔ have been described previously [60].

### 2.2. JCPyV Infection

All cell types were seeded in 96-well plates with ~10,400 cells/well, for 70% confluency at the time of infection. Cells were infected at 37 °C for 1 h with 42 μL/well of MEM containing 10% FBS, 1% P-S, and 0.1% Plasmocin prophylactic, across all cell types. The multiplicities of infection (MOIs) are indicated in the figure legend. Following the 1 h incubation, cells were fed with 100 μL/well of medium and incubated at 37 °C for the duration of the infection. Cells were fixed at timepoints indicated in the legends and either stained for indirect immunofluorescence to determine % infection or stained for ICW to measure protein expression.

### 2.3. Chemical Inhibitor Treatments

All chemical inhibitors were reconstituted in DMSO. Inhibitors that targeted the PI3K/AKT/mTOR signaling pathway were used to pretreat cells for 30 min, while U0126 pretreatment was for 1 h. The concentrations of chemical inhibitors are indicated in the figure legends. All chemical inhibitors were diluted in MEM containing 10% FBS, 1% P-S, and 0.1% Plasmocin prophylactic, prior to JCPyV infection. Following the 1 h viral incubation, all cell types were fed with 100 µL/well of media containing the chemical inhibitor or the DMSO control. For experiments that quantified protein expression, all chemical inhibitors were diluted in incomplete MEM (0% FBS), and cells were treated for 24 h and then fixed in 4% PFA for subsequent analysis. U0126 was purchased from Cell Signaling Technology (Danvers, MA, USA), (#9903S), wortmannin was purchased from Sigma (St. Louis, MO, USA) (#W1628), rapamycin was purchased from Frontier Scientific (Logan, UT, USA (#JK948477), and MK2206 and PP242 was purchased from Selleckchem (Houston, TX, USA) (#S1078 and #S2218). The concentrations of all the chemical inhibitors utilized did not affect cell viability as measured by 4,5-dimethylthiazol-2-yl)-5-(3A-carboxymethoxyphenyl)-2-(4-sulfophenyl)-2H-tetrazolium (MTS) assay (Promega, Madison, WI, USA) (data not shown).

### 2.4. Indirect Immunofluorescence Staining and Quantitation of JCPyV Infection

Following JCPyV infection, all cell types were stained for T Ag or VP1 (Table 1) at RT. When quantifying for JCPyV T Ag, NHAs, SVGAs and NHA-Ts were fixed at 48 hpi, and when quantifying for both viral proteins, cells were fixed at 72 hpi and stained for both JCPyV T Ag and VP1. Cells were fixed with 4% PFA for 10 min and washed with 1 X phosphate-buffered saline (PBS) with 0.01% Tween. NHAs, SVGAs, and NHA-Ts were then permeabilized using PBS-0.5% Triton X-100 for 15 min and blocked with PBS with 0.01% Tween and 10% goat serum for 45 min. Cells were then stained for T Ag or both viral proteins using the antibodies listed in Table 1 at RT for 1 h. Following the 1° antibody incubation, all cell types were washed three times in PBS-0.01% Tween and counterstained with an anti-mouse Alexa Fluor 594 2° antibody at RT for 1 h. Cells were then washed with PBS-0.01% Tween and the nuclei were stained using DAPI (4′,6-diamidino-2-phenylindole) at RT for 5 min. Finally, cells were washed with PBS-0.01% Tween and stored in PBS-0.01% Tween.

To quantify infectivity and nuclear expression of viral protein, T Ag or VP1 over the total number of DAPI-positive cells (percent infection), a Nikon Eclipse Ti epifluorescence microscope (Micro Video Instruments, Inc., Avon, MA, USA) equipped with a 20× objective was used. T Ag- or VP1-expressing cells were counted manually; however, the DAPI-positive cells were determined using a binary algorithm in Nikon NIS-Elements Basic Research software (Micro Video Instruments, Inc.) (version 4.50.00, 64 bit). This algorithm separated cells based on three variables: intensity, diameter, and circularity, resulting in an accurate measurement of the total number of cells in each field of view [31,61,62,63,64].

### 2.5. ICW Assay to Measure Protein Expression Using LI-COR Software

Protein expression measuring phosphorylated ERK, phosphorylated AKT, and phosphorylated mTOR were performed at 24 h post-treatment using an ICW assay [64,65]. Cells were plated in a 96-well plate at ~100% confluency and treated with the various chemical inhibitors for 24 h. Following treatment of the inhibitors, cells were fixed in 4% PFA and washed with PBS-0.01% Tween. Cells were then permeabilized with 1× PBS-0.5% Triton X-100 at RT for 15 min and blocked with Tris-buffered saline (TBS) Odyssey buffer (LI-COR) at RT for 1 h. All cell types were stained with the respective 1° antibody (Table 1) in TBS Odyssey blocking buffer at 4 °C for ~16 h while rocking. The following day, the 1° antibody was removed, and the cells were washed twice with PBS-0.01% Tween. NHAs, SVGAs, and NHA-Ts were then counterstained with 2° antibody, as indicated in Table 1 and CellTag (1:500, LI-COR) for 1 h. Finally, cells were washed with PBS-0.01% Tween and the wells were aspirated. Prior to scanning, the bottom of the plate was cleaned with 70% ethanol and the lid was removed. Plates were weighted with a silicone mat (LI-COR) and imaged using the LI-COR Odyssey CLx infrared imaging system (LI-COR) to detect both the 700 and 800 nm intensities. The imaging settings for the LI-COR were as follows: medium quality, 42 μm resolution, with a 3.0 mm focus offset; when the scan was complete, the 700 and 800 nm channels were aligned and the ICW analysis was performed in Image Studio (version 5.2) (LI-COR). Protein quantification was determined in two steps. First, background fluorescence from the 800 nm channel (wells that only received 2° antibody) were subtracted to the 800 nm channel, in which the protein of interest was being quantified. Next, the ratio was determined using this new value (protein of interest), normalized to the 700 nm channel (CellTag) or the overall number of cells in each well [61,65,66].

### 2.6. RNA-seq and Pathway Analysis

RNA-seq data is available under the accession number: GSE183322. The read counts were generated as previously described [66] and analyzed using RStudio (version 1.2.1335, Boston, MA, USA) and R/edgeR (version 3.30.3) http://bioconductor.org/packages/release/bioc/html/edgeR.html) (accessed on 1 June 2021) [67]. Genes expressed were mapped to the PI3K/AKT signaling pathway from KEGG (https://www.kegg.jp) (accessed on 1 June 2021) [68,69,70] by obtaining the gene symbols and then converting them to Ensembl Gene IDs using EnsemblBioMart (Ensembl version 103, https://www.ensembl.org) (accessed on 1 June 2021) [71]. The Ensembl Gene IDs were matched to the RNA-seq data to acquire the genes expressed in the PI3K/AKT signaling pathway. The list of expressed PI3K/AKT genes was then merged with the results of the R/edgeR analysis [66] and subdivided based on an unadjusted *p* value of less than 0.10. Venn diagrams showing the overlap between the differentially expressed genes for each cell type and timepoint were created using the R package, eulerr (version 6.1.0, https://cran.r-project.org/web/packages/eulerr/) (accessed on 1 June 2021) [72].

### 2.7. Statistical Analysis and Graphing in RStudio

A two-sample Student’s *t* test assuming unequal variances was used to compare the mean values for at least triplicate samples when the data was normally distributed. Non-normal distribution of data was determined by both the Shapiro–Wilk’s test and a quantile-quantile (Q-Q plot) in R. Statistical analyses were performed using the Wilcoxon signed rank test, to compare the median values for two populations, or the Kruskal–Wallis test to compare the median values of more than two populations. If the Kruskal–Wallis test determined a significant result with at least two groups, then the pairwise Wilcoxon rank sum test, along with the Bonferroni adjustment, was used to determine the pairs of groups that were statistically different. The Student’s *t* test was determined in Microsoft Excel (version 2110, Microsoft, Redmond, WA, USA), and all other statistical tests were performed using R. Statistical analyses for the RNA-seq data was performed using R/edge R (version 3.30.3, http://bioconductor.org/packages/release/bioc/html/edgeR.html) (accessed on 1 June 2021) [67].

## 3. Results

### 3.1. U0126, a Common MEK Inhibitor, Does Not Reduce JCPyV Infection in Primary Astrocytes

**Figure 1:**
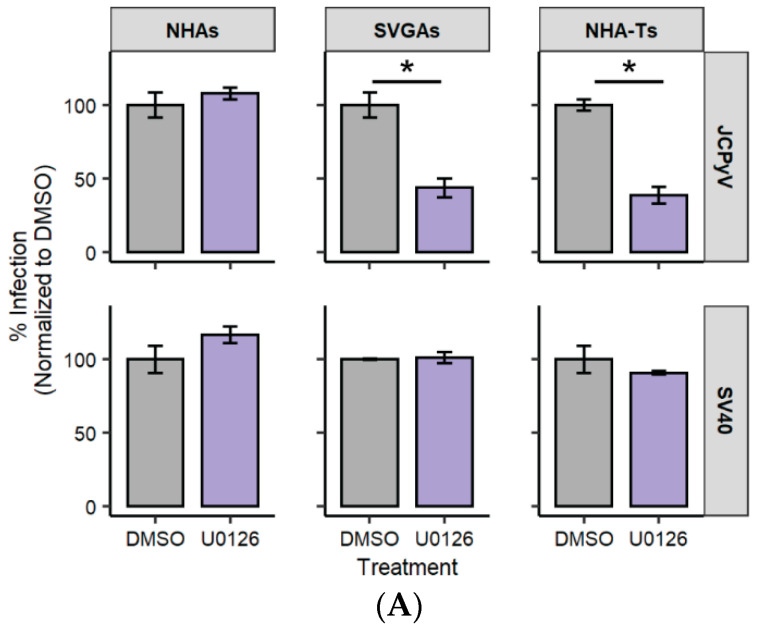

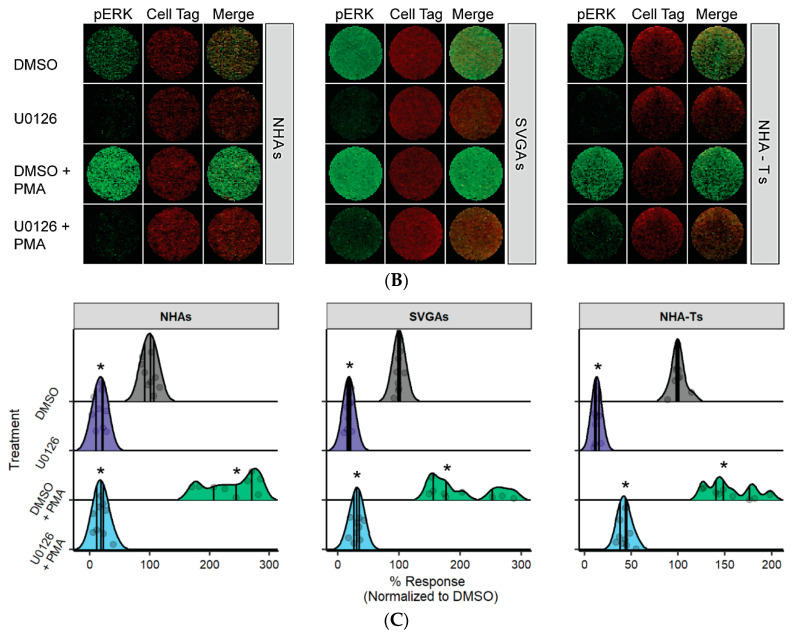
MEK inhibitor U0126 does not decrease JCPyV infection in NHAs. (**A**) NHAs, SVGAs, and NHA-Ts were pretreated with the MEK inhibitor U0126 at 10 µM or DMSO at an equivalent volume control and then infected with JCPyV (MOI = 2.0 FFU/cell; **A**, top) or SV40 (MOI = 2.0 FFU/cell; **A**, bottom) at 37 °C for 1 h. Cells were incubated in media containing DMSO or U0126 for 48 h and then fixed and stained by indirect immunofluorescence. Percent infection was determined by counting the number of JCPyV T Ag- or SV40 VP1-positive nuclei divided by the number of DAPI-positive nuclei for five ×20 fields of view for triplicate samples. Data is representative of three individual experiments. Error bars indicate SD. Student’s *t* test was used to determine statistical significance comparing DMSO to U0126, with each cell type and viral protein. *, *p* < 0.01. (**B**) NHAs, SVGAs, and NHA-Ts were pretreated with DMSO or U0126 for 1 h in addition to PMA (40 nM) treatment of the indicated wells for the final 5 min at 37 °C. Cells were fixed and stained for pERK (green) or CellTag (red). (**C**) Percentage of pERK for each treatment was quantitated by In-Cell Western signal intensity values per [(pERK)/Cell Tag × 100% = % response] within each ICW analysis with LI-COR software (representative image shown in **B**). Density ridgeline plots represent the distribution of samples (individual points) with the lower quartile, median and upper quartile denoted as black lines in each distribution. Colored points represent individual points for each treatment (3 replicates, performed in triplicate). A Kruskal–Wallis test, along with the Bonferroni adjustment, was used to compare treatments in each cell type; however, only significance with the DMSO treatment versus other treatments are illustrated. *, *p* < 0.01. Data are representative of three independent experiments performed in triplicate.

In addition to the PI3K/AKT/mTOR signaling pathway implicated in JCPyV infection [48,50,59], the mitogen-activated protein kinase, extracellular signal-regulated kinase (MAPK/ERK) pathway is required for JCPyV infection [61,73,74]. This pathway is temporally regulated, specifically being phosphorylated upon JCPyV infection, and infection of immortalized cells is significantly reduced when cells are treated with ERK siRNAs or inhibitors [61,66,73,74]. The MAPK/ERK pathway overlaps with the PI3K/AKT/mTOR pathway and both pathways have important roles in cell survival and differentiation [39,75]. To determine if inhibiting the phosphorylation of ERK decreases JCPyV in primary astrocytes, a well-known MEK inhibitor, U0126, was tested for its effects on JCPyV infection. NHAs, SVGAs, and NHA-Ts were treated with U0126 at 10 µM or the DMSO vehicle control (vehicle control), and subsequently infected with JCPyV (Figure 1A). U0126 did not decrease infection in NHAs at 48 hpi compared to SVGAs and NHA-Ts (Figure 1A, top). As a negative control, all cell types were infected with SV40, which does not require MEK for infection [76,77], and U0126 did not influence SV40 infection in any cell type (Figure 1A, bottom). To confirm the inhibitory effect of U0126 in NHAs, SVGAs, and NHA-Ts, ERK phosphorylation following treatment of the MEK inhibitor for 1 h was evaluated by In-Cell Western (ICW; Figure 1B). U0126 nearly abolished ERK phosphorylation in all three cell types (Figure 1C). PMA, an ERK activator, was used as a control to further determine the specificity of the MEK inhibitor. All cell types were treated with U0126 for 1 h, treated with PMA for 5 min, and ERK phosphorylation was nearly reduced to comparable levels to cells treated with U0126 alone (Figure 1C). Together, these data suggest that although elevated levels of ERK phosphorylation promote JCPyV infection [66], inhibition of ERK phosphorylation does not decrease JCPyV infection in primary astrocytes when compared to cells immortalized with SV40 T Ag. Thus, these data suggest that the mechanisms of MAPK cell signaling activation utilized in JCPyV infection of NHAs differs from those in SVGAs and NHA-Ts.

### 3.2. AKT Phosphorylation Is Moderately Increased in NHAs during U0126 Treatment Compared to SVGAs and NHA-Ts

**Figure 2:**
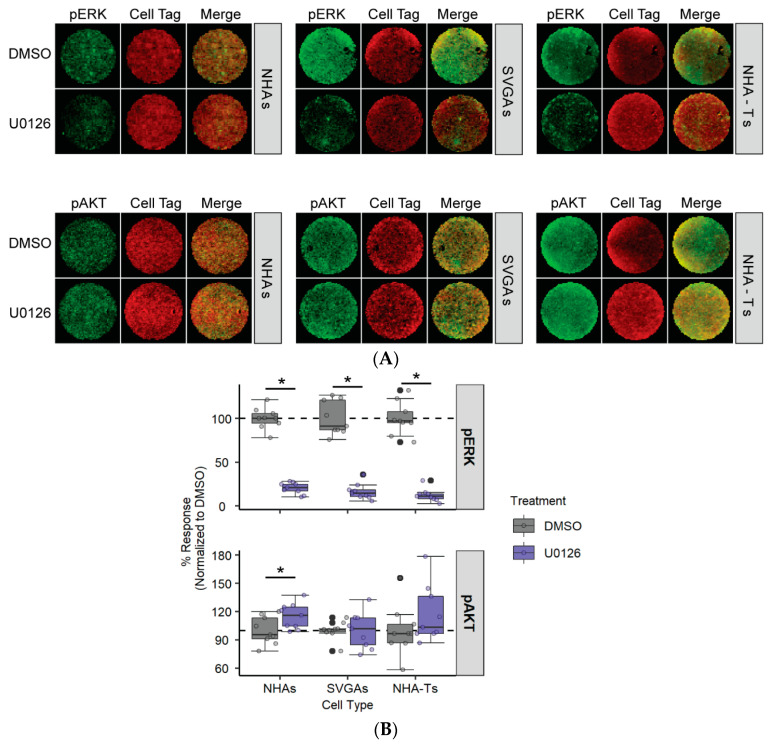
U0126 increases AKT phosphorylation in NHAs. (**A**) NHAs, SVGAs, and NHA-Ts were treated with DMSO or U0126 (10 µM) at 37 °C for 24 h. Cells were fixed and stained for pERK or pAKT (green) or CellTag (red). Entire wells of a 96-well plate are shown (**B**) Percentage of pERK and pAKT for each cell type was quantitated by ICW signal intensity values per [(pERK or pAKT)/Cell Tag × 100% = % response] within each ICW analysis with LI-COR software. Box and whisker plots represent the distribution of samples (individual points), with the lower quartile, median and upper quartile denoted as black lines. Colored points represent individual points for each cell type (3 replicates, performed in triplicate), and outliers are represented by black circles. The dashed line indicates the normalized DMSO % response of pERK and pAKT. A Wilcoxon rank sum exact test was used to compare treatments in each cell type. Data are representative of three independent experiments performed in triplicate. *, *p* < 0.05.

The ERK signaling cascade can interact with the AKT signaling pathway [78] and they work together to induce cellular transformation and survival [39,75]. Research has demonstrated that treatment of cells with MEK inhibitors, such as U0126, results in increased AKT activity in various cell types [79,80,81]. To determine if U0126 influences AKT phosphorylation, NHAs, SVGAs, and NHA-Ts were treated with U0126 and AKT phosphorylation was measured (Figure 2). Cells were serum-starved and treated with U0126 (10 µM) or the vehicle control for 24 h, and both ERK and AKT phosphorylation were measured by ICW (Figure 2A). U0126 increased AKT phosphorylation in NHAs, albeit by ~20%, while AKT phosphorylation was not altered in SVGAs and NHA-Ts (Figure 2B). This experiment demonstrated that the crosstalk between the AKT signaling pathway and the ERK signaling cascade occurs in primary astrocytes. Additionally, it provides a premise for explaining why U0126 did not decrease JCPyV infection in NHAs; U0126 inhibits ERK phosphorylation, suggesting that the virus may also hijack the AKT signaling pathway to support viral replication.

### 3.3. PI3K/AKT Signaling Pathway Genes Are Upregulated during JCPyV Infection in NHAs

**Figure 3:**
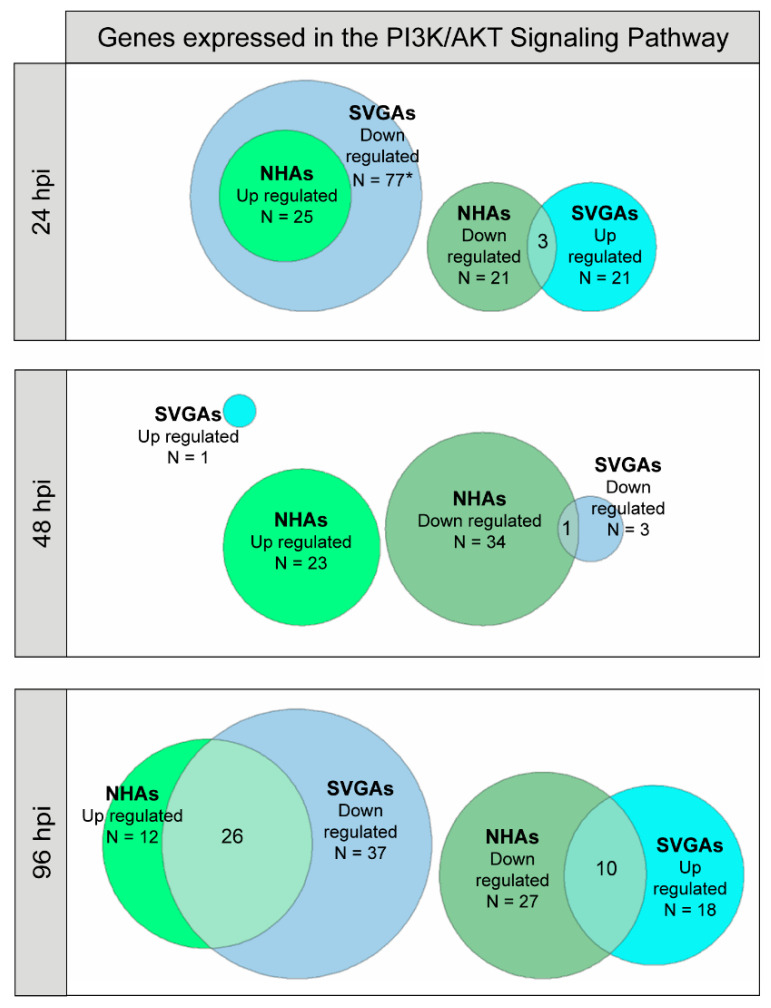
Genes in the PI3K/AKT pathway were differentially expressed during JCPyV infection in NHAs compared to viral infection in SVGAs. NHAs and SVGAs were either infected with JCPyV (MOI = 0.1 FFU/cell) or mock-infected with a vehicle control, and the transcriptomic profile was determined at 24, 48, and 96 h using RNA-seq. Genes of the PI3K/AKT pathway were determined from the KEGG database with an unadjusted *p* value < 0.10. Upregulated genes (Log FC > 0) and downregulated genes (Log FC < 0) are represented as Venn diagrams for each time point and cell type. The size of each circle is proportionate to the number of genes that apply to the above criteria. The green colors are representative of NHAs, while the blue colors are representative of SVGAs. Brighter shades represent upregulated genes and darker shades represent downregulated genes. (FC, fold-change; * 77 genes in total are down regulated in SVGAs, but 52 genes are exclusively downregulated in SVGAs.).

Previous research has demonstrated that the PI3K/AKT signaling pathway, specifically the AKT and PI3Kγ steps, is required for JCPyV infection in SVGAs and an oligodendrocyte cell line [48,59,82]. To determine if this pathway is implicated in JCPyV infection in NHAs, RNA-seq data was analyzed [66] over the course of JCPyV infection in NHAs and SVGAs. Differentially-expressed genes (unadjusted *p* value < 0.10) were mapped to the PI3K/AKT signaling pathway from the Kyoto Encyclopedia of Genes and Genomes (KEGG) database [68,69,70]. The table of genes with their *p* values and log-fold changes (FCs) can be found in the Supplementary Material (Table S1). At 24 hpi, 25 genes that mapped to the pathway were downregulated in SVGAs but upregulated in NHAs (Figure 3, top). At 48 hpi, only one gene was upregulated in SVGAs compared to 23 genes upregulated in NHAs (Figure 3, middle). At 96 hpi, approximately 51% of the genes that mapped to the pathway were upregulated in NHAs versus ~38% in SVGAs (Figure 3, bottom). These data illustrate that compared to SVGAs, JCPyV infection in NHAs activated more genes in the PI3K-AKT signaling pathway, providing additional evidence for the involvement of this pathway during infection in primary astrocytes.

### 3.4. AKT Is Differentially Expressed and Required for JCPyV Infection in NHAs

**Figure 4:**
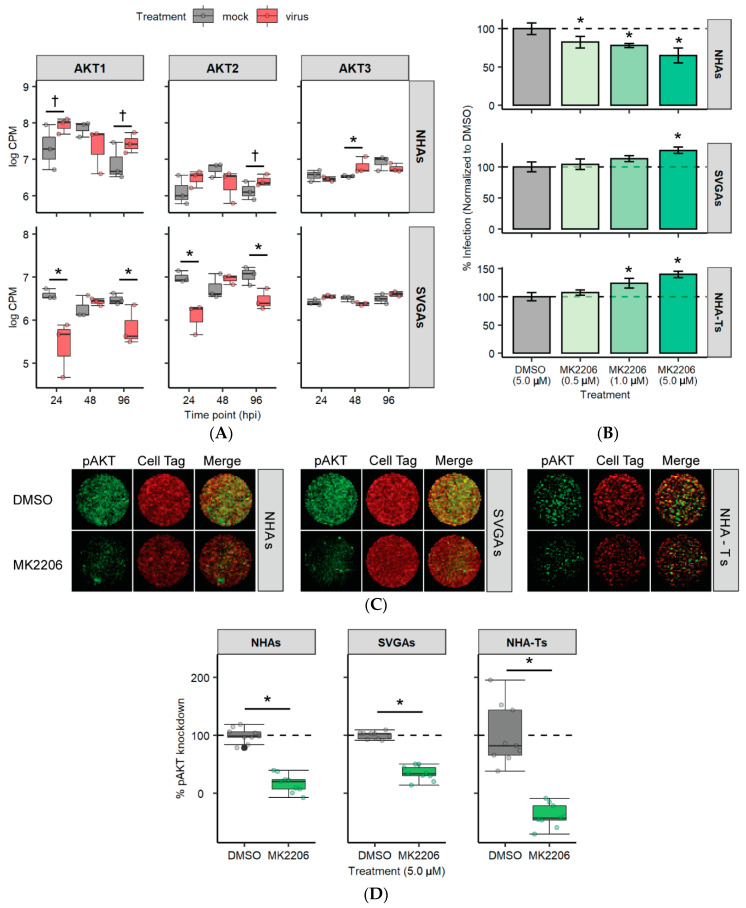
AKT is required for JCPyV infection in NHAs. (**A**) The log CPM was determined for the three isoforms of AKT (*AKT1*, *AKT2*, and *AKT3*) in mock-infected and JCPyV-infected cells at each time point from the RNA-seq data. Colored points represent individual points for each timepoint (3 replicates). †, *p* < 0.10; *, *p* < 0.05. (**B**) NHAs, SVGAs, and NHA-Ts were pretreated with indicated concentrations of AKT inhibitor MK2206 or DMSO at an equivalent volume control for 30 min and then infected with JCPyV (MOI = 1.0 FFU/cell) at 37 °C for 1 h. Cells were incubated in media containing DMSO or MK2206 for 48 h and then fixed and stained by indirect immunofluorescence. Infectivity was determined by counting the number of JCPyV T Ag-positive nuclei divided by the number of DAPI-positive nuclei for five ×20 fields of view for triplicate samples (% infection). Data is representative of three individual experiments. Error bars indicate SD. Student’s *t* test was used to determine statistical significance, comparing DMSO to MK2206 for each cell type. (**C**) NHAs, SVGAs, and NHA-Ts were treated with DMSO or MK2206 (5.0 µM) at 37 °C for 24 h. Cells were fixed and stained for pAKT (green) or CellTag (red). (**D**) Percentage of pAKT for each cell type was quantitated by ICW signal intensity values per [(pAKT)/Cell Tag × 100% = % response] within each ICW analysis using LI-COR software (representative image shown in **C**). Box and whisker plots represent the distribution of samples (individual points) with the lower quartile, median, and upper quartile denoted as black lines. The dashed line indicates the normalized DMSO % response of pAKT. Colored points represent individual points for each treatment (3 replicates, performed in triplicate), and outliers are represented by black circles. A Wilcoxon rank sum exact test was used to compare treatments in each cell type. Data are representative of three independent experiments performed in triplicate. * *p* < 0.05.

To further validate the previous results, the expression of the three isoforms of AKT (*AKT1*, *AKT2* and *AKT3*) were analyzed (Figure 4A). Both *ATK1* and *ATK2* were significantly downregulated in SVGAs at 24 and 96 hpi (unadjusted *p* value < 0.05) and upregulated in NHAs (unadjusted *p* value < 0.1; Figure 4A). Due to the differential gene expression observed, NHAs, SVGAs, and NHA-Ts were pretreated with the AKT inhibitor, MK2206, or the vehicle control at increasing concentrations, and subsequently infected with JCPyV (Figure 4B). A dose-dependent decrease in viral infection was observed in NHAs; however, a dose-dependent increase was measured in SVGAs and NHA-Ts (Figure 4B). Considering the significant differences in JCPyV infection among cell types, MK2206 was validated for its inhibitory effects on AKT phosphorylation by ICW (Figure 4C). Cells were serum-starved and treated with the highest concentration of MK2206 for 24 h. The chemical inhibitor significantly reduced AKT phosphorylation in all three cell types compared to the vehicle control, demonstrating that the inhibitor is working effectively at the concentrations used (Figure 4D). This data revealed that JCPyV requires AKT during infection in NHAs and the utilization of this pathway is perhaps confounded by the immortalized properties of SVGAs and NHA-Ts.

### 3.5. PI3K Is Required for JCPyV Infection in NHAs

**Figure 5:**
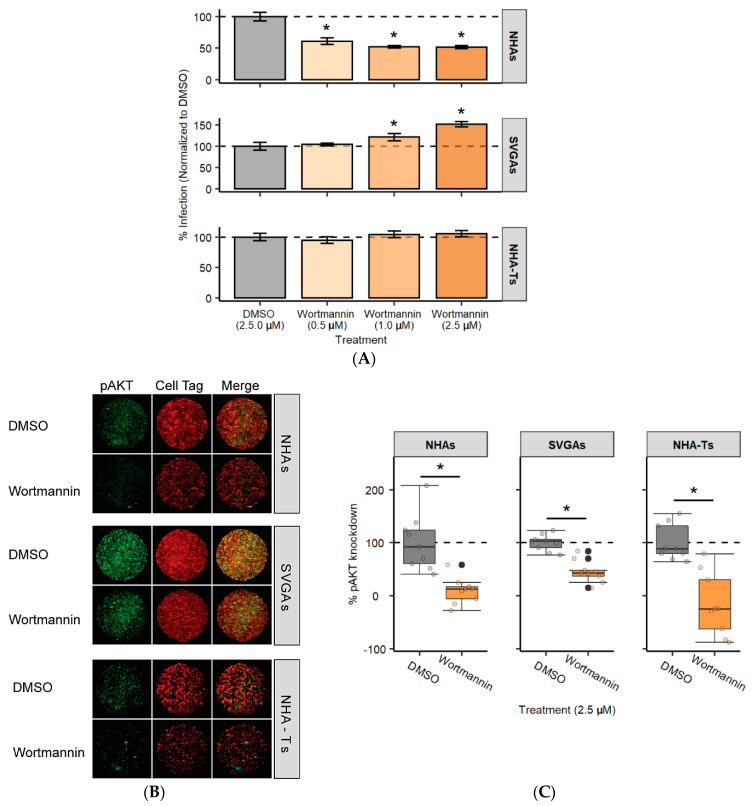
PI3K inhibitor wortmannin reduces JCPyV infection in NHAs. (**A**) NHAs, SVGAs, and NHA-Ts were pretreated with indicated concentrations of a PI3K inhibitor, wortmannin, or DMSO at an equivalent volume control and then infected with JCPyV (MOI = 1.0 FFU/cell) at 37 °C for 1 h. Cells were incubated in media containing DMSO or wortmannin for 48 h and then fixed and stained by indirect immunofluorescence. Infectivity was determined by counting the number of JCPyV T Ag-positive nuclei divided by the number of DAPI-positive nuclei for five ×20 fields of view for triplicate samples (% infection). Data is representative of three individual experiments. Error bars indicate SD. Student’s *t* test was used to determine statistical significance comparing DMSO to wortmannin for each cell type. (**B**) NHAs, SVGAs, and NHA-Ts were treated with DMSO or wortmannin (2.5 µM) at 37 °C for 24 h. Cells were fixed and stained for pAKT (green) or CellTag (red). Entire wells of a 96-well plate are shown. (**C**) Percentage of pAKT for each cell type was quantitated by ICW signal intensity values per [(pAKT)/Cell Tag × 100% = % response] within each ICW analysis using LI-COR software (representative image shown in B). Box and whisker plots represent the distribution of samples (individual points) with the lower quartile, median and upper quartile denoted as black lines. The dashed line indicates the normalized DMSO % response of pAKT. Colored points represent individual points for each treatment (3 replicates, performed in triplicate), and outliers are represented by black circles. A Wilcoxon rank sum exact test was used to compare treatments in each cell type. Data are representative of three independent experiments performed in triplicate. *, *p* < 0.05.

Upstream of AKT, PI3K was targeted using a chemical inhibitor to understand if additional proteins in the PI3K/AKT pathway are involved during JCPyV infection in NHAs. Cells were pretreated with the PI3K inhibitor, wortmannin, or the vehicle control at increasing concentrations, and subsequently infected with JCPyV (Figure 5A). Comparable to AKT inhibition, wortmannin significantly reduced JCPyV infection by 50% in NHAs, while increasing infection in SVGAs and not influencing infection in NHA-Ts (Figure 5A). The chemical inhibitor was validated for its inhibition of the PI3K/AKT signaling pathway by measuring AKT phosphorylation using ICW (Figure 5B). Cells were serum-starved and treated with the highest concentration of wortmannin. Across all three cell types, wortmannin significantly reduced AKT phosphorylation (Figure 5C). Altogether, these data illustrate that treatment of cells with the PI3K inhibitor equivalently impaired the PI3K/AKT signaling pathway, and a decrease in JCPyV infection during PIK3K inhibition was only observed in NHAs, verifying the results demonstrated with MK2206 treatment (Figure 4).

### 3.6. mTOR Inhibition Significantly Reduces JCPyV Infection in NHAs

**Figure 6:**
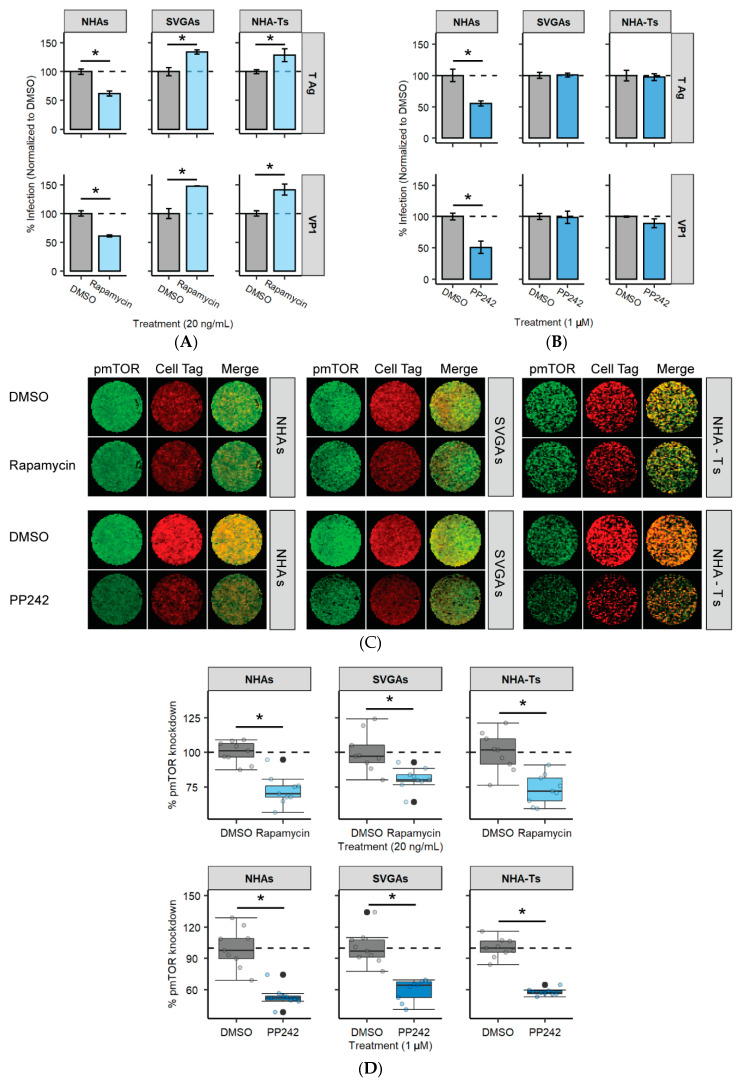
Inhibitors of mTOR, a target of the PI3K/AKT pathway, reduce JCPyV infection in NHAs. (**A**,**B**). NHAs, SVGAs, and NHA-Ts were pretreated with (**A**) rapamycin at 20 ng/mL, (**B**) PP242 at 1 µM, or the DMSO control at an equivalent volume control and then infected with JCPyV (MOI = 1.0 FFU/cell) at 37 °C for 1 h. Cells were incubated in media containing DMSO or mTOR inhibitors for 72 h and then fixed and stained by indirect immunofluorescence. Infectivity was determined by counting the number of JCPyV T Ag- or VP1- positive nuclei divided by the number of DAPI-positive nuclei for five ×20 fields of view for triplicate samples (% infection). Data is representative of three individual experiments performed in triplicate. Error bars indicate SD. Student’s *t* test was used to determine statistical significance comparing DMSO to either mTOR inhibitor, with each cell type and viral protein. (**C**) All cell types were treated with DMSO or rapamycin at 20 ng/mL (top) or PP242 at 1 µM (bottom) at 37 °C for 24 h. Cells were fixed and stained for pAKT (green) or CellTag (red). Entire wells of a 96-well plate are shown. (**D**) Percentage of pmTOR for each cell type was quantitated by ICW signal intensity values per [(pmTOR)/Cell Tag × 100% = % response] within each ICW analysis with LI-COR software (representative image shown in **C**). Box and whisker plots represent the distribution of samples (individual points) with the lower quartile, median, and upper quartile denoted as black lines. The dashed line indicates the normalized DMSO % response of pmTOR. Colored points represent individual points for each treatment (3 replicates, performed in triplicate), and outliers are represented by black circles. A Wilcoxon rank sum exact test was used to compare treatments in each cell type. Data are representative of three independent experiments performed in triplicate. *, *p* < 0.05.

A downstream target of the PI3K/AKT signaling pathway is mTOR [83], and previous research has determined that mTOR inhibition can increase JCPyV infection in an immortalized kidney cell line [50]. Moreover, our previous research has also demonstrated that JCPyV infection is delayed in NHAs, compared to SVGAs and NHA-Ts [31]. To determine if mTOR is required for JCPyV infection and if mTOR inhibition increases late viral protein expression in NHAs, all cell types were treated with either rapamycin, mTOR inhibitor PP242, or the vehicle control, and were subsequently infected with JCPyV, and infectivity was measured by assessing both early (T Ag) and late (VP1) viral protein production (Figure 6). Rapamycin and PP242 significantly reduced JCPyV infection in NHAs as quantified by both T Ag and VP1 production (Figure 6A,B). Furthermore, rapamycin significantly increased JCPyV infection in SVGAs and NHA-Ts (Figure 6A). However, PP242 did not influence viral infection in either SVGAs or NHA-Ts (Figure 6B). To validate the effectiveness of the chemical inhibitors in NHAs, SVGAs, and NHA-Ts, mTOR phosphorylation was measured by ICW, following serum starvation and treatment with each inhibitor for 24 h (Figure 6C). Rapamycin and PP242 significantly reduced mTOR phosphorylation, relatively, in each cell type; however, PP242 reduced phosphorylation to more appreciable levels when compared to rapamycin (Figure 6D). Overall, these findings demonstrate that mTOR phosphorylation, a downstream target of the PI3K/AKT signaling pathway, is required for JCPyV infection in NHAs. Furthermore, it substantiates previous findings that JCPyV uses alterative signaling pathways in primary astrocytes—viral mechanisms that are not observed in cell types that are immortalized with SV40 T Ag.

## 4. Discussion

Astrocytes are the main targets of JCPyV infection in the CNS, where the destruction of these cells, along with oligodendrocytes, leads to PML [1,2,3,4,26]. With no cure for this aggressive and ultimately fatal disease, more research is needed to reveal potential therapeutic targets. Cellular pathways, like the PI3K/AKT/mTOR signaling pathway, is an attractive candidate because there are numerous established drugs that inhibit this pathway for cancer treatment [51,52]. Additionally, there is evidence to suggest that these drugs can be repurposed to target coronaviruses, such as SARS-CoV-2 [84,85,86,87], the causative agent of COVID-19 [88]. In this study, we investigated the role of various chemical inhibitors of the PI3K/AKT/mTOR signaling pathway on JCPyV infection in primary astrocytes compared to immortalized cells. Our results demonstrate that viral infection is significantly reduced in primary astrocytes compared to cells transformed with SV40 T Ag, highlighting both the importance of this signaling pathway, and the need to use either ex vivo approaches or to validate findings in primary cells to further understand how JCPyV infection may occur in the human host.

JCPyV requires the MAPK/ERK pathway to successfully infect immortalized cells [61,66,73,74]. However, when a well-studied MEK inhibitor, U0126, did not reduce JCPyV infection in primary astrocytes, yet reduced JCPyV infection in cells immortalized with SV40 T Ag (Figure 1), additional studies were performed to identify alternative pathways that regulate JCPyV infection. Research has shown that the PI3K/AKT/mTOR signaling pathway intersects with the MAPK/ERK pathway [79,80,81]. Our findings also support this, as treatment of NHAs with U0126 led to an increase in phosphorylated AKT, while U0126 treatment did not exhibit any difference in AKT phosphorylation in SVGAs and NHA-Ts (Figure 2).

The PI3K/AKT/mTOR signaling pathway has been previously implicated in other polyomavirus infections as well [48,50,59,89,90,91,92]. Earlier research has demonstrated that the BK polyomavirus (BKPyV), murine polyomavirus, and JCPyV influence the PI3K/AKT signaling pathway by modulating the cellular phosphatase, protein phosphatase 2A (PP2A) [89,90,91]. Comparable to JCPyV, BKPyV also establishes an asymptomatic infection in the kidney, yet during immunosuppression BKPyV can cause nephropathy and hemorrhagic cystitis [93]. Treatment of cells with sirolimus (i.e., rapamycin) significantly reduces BKPyV infection; however, the concentration of the chemical inhibitor used in those studies was significantly different between immortalized and primary cells [89,92]. Hirsch et al. used a primary kidney cell line to determine the outcome of BKPyV infection during rapamycin treatment. They confirmed that using rapamycin at least a magnitude lower in concentration significantly reduces BKPyV infection in a primary kidney cell line, specifically during the first 24 h of infection (i.e., before viral genome replication) [92]. The authors concluded that these differences in concentration of the drug were the result of the transformed phenotype of the other cell line, causing it to require significantly higher concentrations of the inhibitor to reduce BKPyV infection. Unfortunately, understanding viral infection is challenging when using transformed cell lines, as transformation alters metabolic pathways and signal transduction within those pathways [94,95].

Our findings substantiate the consequences of immortalization in investigations of JCPyV infection with chemical inhibitors that target the PI3K/AKT/mTOR signaling pathway. Wortmannin, MK2206, rapamycin, and PP242—chemical inhibitors that target different steps of PI3K/AKT/mTOR signaling pathway—resulted in a significant reduction of JCPyV infection in NHAs that was not observed in SVGAs or NHA-Ts. These results, specifically with MK2206, are consistent with other published research reporting the impacts of JCPyV infection on an oligodendrocyte cell line treated with inhibitors of this pathway. MK2206 treatment of a glioma-derived stem cell line with oligodendrocyte precursor phenotypes (G144 cells) reduced JCPyV DNA replication [59]. It is important to note, that even though these cells are established through glioblastoma samples, G144 cells specifically display features that resemble normal fetal neural stem cells [96]. Together, these data suggest the importance of MK2206 as a potential antiviral for PML, as oligodendrocytes and astrocytes are the main cell types impacted by disease [1,2,3,4,26].

Our research also corresponds with findings demonstrated with BKPyV infection and mTOR inhibition with respect to both polyomavirus infection and cell-type dependent differences. Rapamycin significantly reduced JCPyV infection in primary astrocytes (Figure 6A), and similarly, BKPyV infection was reduced in a primary kidney cell line with rapamycin treatment [92]. Likewise, using both rapamycin and PP242—a secondary mTOR inhibitor—did not decrease JCPyV infection in SVGA and NHA-T immortalized cells (Figure 6A,B). Similarly, rapamycin and other mTOR inhibitors did not decrease but rather enhanced JCPyV replication in HEK293A cells [50]. HEK293A cells are transformed with adenovirus type 5 DNA [97], but not with SV40 T Ag; yet, immortalization through adenovirus leads to adenovirus oncogene E1A interactions with Rb and p53, disrupting important checkpoints in cell cycle and growth, similar to SV40 T Ag transformation [98,99,100,101,102]. Additionally, cells transformed with viral oncogenes can also influence PP2A, known to regulate numerous cellular pathways, such as the MAPK and PI3K signaling pathways—which are also important during JCPyV infection [91,103,104]. Together, these findings demonstrate both the requirement for the PI3K/AKT/mTOR signaling pathway and the importance of using primary cell lines to characterize polyomavirus infection.

Furthermore, rapamycin and PP242 are both mTOR inhibitors, yet the mode of inhibition is slightly different. mTOR forms two complexes in mammalian cells—mTOR complex 1 (mTORC1) and mTORC2—and activation of these complexes results in different functions for the cell [105]. The formation and activation of mTORC1 results in protein translation, cell growth, and autophagy, while mTORC2 results in survival, migration, and cytoskeletal organization [105]. Rapamycin has been demonstrated to inhibit mTORC1 more so than mTORC2—particularly in vitro [106]—while PP242 results in greater inhibition of both mTORC1 and mTORC2 [107]. These modes of mTOR inhibition may explain the differences observed in immortalized cells during JCPyV infection; however, it does substantiate the importance of mTOR during infection of primary astrocytes (Figure 6). Additionally, if the concentration of PP242 was increased, the results of JCPyV infection were similar. In fact, higher concentrations resulted in cytotoxic effects (data not shown), yet each chemical inhibitor was tested for cellular viability compared to the vehicle control in each cell type, and concentrations well-tolerated by cells were used in all the assays performed. Additionally, the inhibitory effect, measuring phosphorylation of AKT or mTOR, was similar across cell types—demonstrating that cell-type differences were not from cytotoxic effects or from inequitable impacts of AKT or mTOR phosphorylation from the chemical treatments.

Research has demonstrated that enhanced JCPyV infection from mTOR inhibition is perhaps due to the Skp-Cullin Fbox (SCF) E3 ligase, S-phase kinase-associated protein 2 (Skp2) [50,108]; the expression of this protein is highly variable in immortalized versus primary cells [109]. Skp2 is important in regulating the cell cycle, accumulates in the cell during the transition to the G1/S phase, remains highly expressed during S phase [110] and can also interact with polyomavirus Large T Ag [50,108]. Previous studies have concluded that the interaction of Skp2 and Large T Ag of numerous polyomaviruses (PyVs), including murine PyV, JCPyV, and BKPyV, was reduced with treatment with mTOR inhibitors, which resulted in an increase of Large T Ag expression [50]. Skp2 is highly expressed in glioma cell lines compared to normal astrocyte cell lines [109]; this could explain the differences observed between NHAs versus SVGAs and NHA-Ts. First, JCPyV Large T Ag expression is significantly lower in NHAs compared to SVGAs and NHA-Ts [31], and as a result, Skp2 may not be involved to the same extent in JCPyV infection of NHAs; thus, this interaction between Skp2 and Large T Ag is not sensitive to mTOR inhibition. However, future research should elucidate the mechanisms of viral protein production and the PI3K/AKT/mTOR signaling pathway.

**Figure 7:**
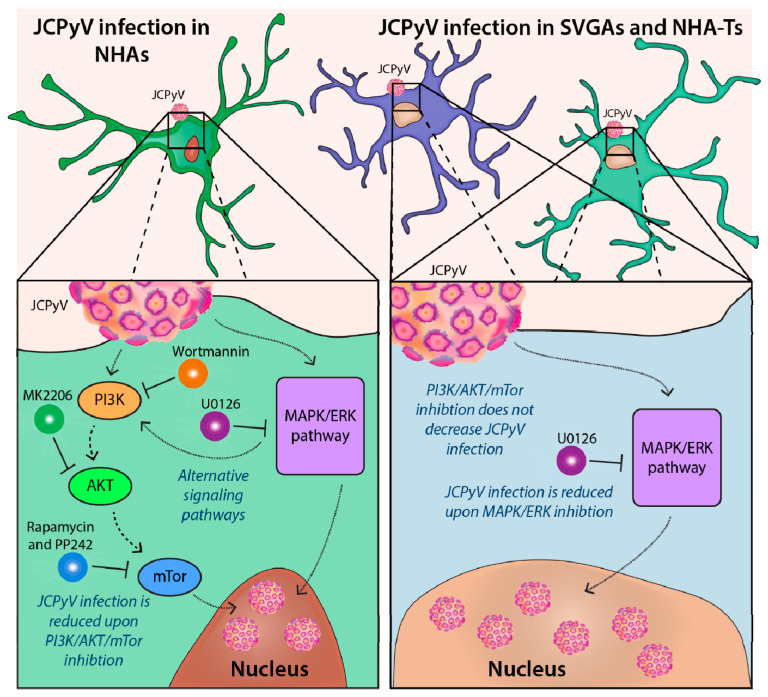
Chemical inhibitors that reduce JCPyV infection in NHAs, SVGAs, and NHA-Ts. Previous research has demonstrated the importance of the MAPK/ERK pathway in NHAs; however, JCPyV can also use the PI3K/AKT/mTOR signaling pathway for infection (**left**). Using chemical inhibitors targeting PI3K, AKT, and mTOR significantly reduced JCPyV infection in primary astrocytes (**left**). However, in immortalized cells, SVGAs and NHA-Ts, these chemical inhibitors did not reduce JCPyV infection and treatment of cells with U0126—an MEK inhibitor of the MAPK/ERK pathway—which significantly reduced JCPyV infection (**right**). This demonstrates that during the viral infection of immortalized cells, JCPyV is more dependent on the MAPK/ERK pathway and may not use other pathways, such as the PI3K/AKT/mTOR signaling pathway, to establish successful infection, compared to JCPyV infection in primary astrocytes.

Lastly, PI3K expression has been recently demonstrated to decrease JCPyV infection in SVGAs [48]. A reason for the differences in JCPyV infection between the findings reported here and by Clark et. al could be the PI3K isoform that was targeted, as well as the technique used. The authors determined that PI3K, specifically PI3Kγ, facilitates JCPyV infection in SVGAs through genetic knockdown approaches [48]. It is known that JCPyV facilitates entry into the cell through the utilization of a GPCR—the serotonin 5-hydroxytryptamine (5-HT_2_) receptor [62,63,111]—which upon activation couples with PI3Kγ [48]. The authors speculated that knockdown of PI3Kγ disrupted early events of GPCR signaling, and as a result, disrupted possible virus capsid disassembly or trafficking to the endoplasmic reticulum or nucleus [48]. Wortmannin is one of the most well-characterized PI3K inhibitors and has been shown to interact strongly in vitro with PI3K, thus inhibiting numerous isoforms in the PI3K family [112,113]. However, wortmannin has also been demonstrated to have off-target effects, inhibiting other serine/threonine kinases of the PI3K family such as mTOR [114]. The differences that we have observed could be the consequence of wortmannin targeting other PI3Ks in the pathway and thus having similar results to the other inhibitors used in this research, specifically in SVGAs. Furthermore, JCPyV entry into primary astrocytes has not been extensively studied, and thus, we do not yet know whether GPCRs are utilized during entry, thereby activating PI3Kγ to the same extent as it is with viral entry of SVGAs. Additionally, recent work has demonstrated that JCPyV can use extracellular vesicles to infect SVGAs and astrocytes independently of the sialic acid attachment receptor through clathrin-dependent and independent mechanisms [115]. Future studies should define the role of PI3Ks in the viral infection of primary astrocytes using more targeted approaches such as siRNA, as well as in primary kidney cells. Nonetheless, wortmannin, MK2206, rapamycin, and PP242 significantly reduced JCPyV infection in primary astrocytes, while also inhibiting the phosphorylation of AKT and mTOR in all three cell types (Figure 7). Furthermore, RNA-seq analysis revealed numerous genes within the PI3K/AKT/mTOR pathway that were upregulated during JCPyV infection of NHAs, but downregulated in SVGAs, providing more evidence of the requirement of this pathway in primary astrocytes (Figure 3 and Figure 4A). The genes β3 integrin (*ITGB3*) and interleukin-6 receptor (*IL-6R*) had a 3.4- and 2-fold increase, respectively, at 96 hpi in NHAs during JCPyV infection (Table S1). ITGB3 is a regulator of the PI3K/AKT/mTOR pathway [116] and has roles in cancer [116,117] and extracellular vesicles that induce cell signaling [118,119]. Interestingly, research has demonstrated that ITGB3 can regulate expression of matrix metalloproteinase 2 (MMP2) [120], a protein that is critical in the inflammatory response and in demyelinating diseases, such as MS [121,122,123]. Overall, this data illustrates how JCPyV activates the PI3K/AKT/mTOR pathway, yet this may have other implications in the viral-induced demyelinating disease PML. More research should determine if astrocytes induce an inflammatory response from activation of the PI3K/AKT/mTOR pathway.

In summary, this research has revealed and outlined the requirement of the PI3K/AKT/mTOR signaling pathway in JCPyV infection of primary human astrocytes. Using various chemical inhibitors, we have characterized how JCPyV uses this pathway to support viral infection in primary cells, and importantly, how immortalized characteristics may alter signaling events that, in turn, confound the requirement of this pathway in JCPyV infection of transformed cell lines. Overall, these findings will aid in the discovery of therapeutics to treat or slow the progression of PML, as no effective treatments are available for this fatal disease.

## Figures and Tables

**Table 1:** Antibodies used in immunofluorescence and ICW assays.

Protein	1° Antibody (Dilution, Source)	2° Antibody (Dilution, Manufacturer)
JCPyV T Ag	PAB962 (1:5, hybridoma, Tevethia Lab, Penn State University, State College, PA, USA)	anti-mouse Alexa Fluor 594 (1:1000, Thermo Fisher Scientific (Waltham, MA, USA))
JCPyV VP1	Ab34756 (1:1000, Abcam)	
pERK (P-p44/42 MAPK at T202/Y204)	9101S (1:750, CST)	anti-rabbit IRDye 800CW (1:10,000, LI-COR, Lincoln, NE, USA)
pAKT (S473)	4060S (1:400, CST)	
pmTOR (S2448)	44-1125G (1:1000, Invitrogen, Waltham, MA, USA)	
CST, Cell Signaling Technology; ICW, In-Cell Western assay		

## Data Availability

The RNA-seq data [66] analyzed in this study is deposited in the National Center for Biotechnology Information (NCBI) Gene Expression Omnibus (GEO) database, under accession number GSE183322.

## Supplementary Materials

The following are available online at https://www.mdpi.com/article/10.3390/cells10113218/s1, Table S1: Log fold change of genes within the PI3K/AKT signaling pathway during JCPyV infection of NHAs and SVGAs.

## References

[1] Hirsch H.H., Kardas P., Kranz D., Leboeuf C. (2013). The Human JC Polyomavirus (JCPyV): Virological Background and Clinical Implications. APMIS.

[2] Padgett B.L., Walker D.L., ZuRhein G.M., Eckroade R.J., Dessel B.H. (1971). Cultivation of Papova-like Virus from Human Brain with Progressive Multifocal Leucoencephalopathy. Lancet.

[3] Silverman L., Rubinstein L.J. (1965). Electron Microscopic Observations on a Case of Progressive Multifocal Leukoencephalopathy. Acta Neuropathol..

[4] Zurhein G., Chou S.M. (1965). Particles Resembling Papova Viruses in Human Cerebral Demyelinating Disease. Science.

[5] Kean J.M., Rao S., Wang M., Garcea R.L. (2009). Seroepidemiology of Human Polyomaviruses. PLoS Pathog..

[6] Egli A., Infanti L., Dumoulin A., Buser A., Samaridis J., Stebler C., Gosert R., Hirsch H.H. (2009). Prevalence of Polyomavirus BK and JC Infection and Replication in 400 Healthy Blood Donors. J. Infect. Dis..

[7] Monaco M.C., Atwood W.J., Gravell M., Tornatore C.S., Major E.O. (1996). JC Virus Infection of Hematopoietic Progenitor Cells, Primary B Lymphocytes, and Tonsillar Stromal Cells: Implications for Viral Latency. J. Virol..

[8] Monaco M.C.G., Jensen P.N., Hou J., Durham L.C., Major E.O. (1998). Detection of JC Virus DNA in Human Tonsil Tissue: Evidence for Site of Initial Viral Infection. J. Virol..

[9] Dubois V., Dutronc H., Lafon M.E., Poinsot V., Pellegrin J.L., Ragnaud J.M., Ferrer A.M., Fleury H.J. (1997). Latency and Reactivation of JC Virus in Peripheral Blood of Human Immunodeficiency Virus Type 1-Infected Patients. J. Clin. Microbiol..

[10] Chapagain M.L., Nerurkar V.R. (2010). Human Polyomavirus JC (JCV) Infection of Human B Lymphocytes: A Possible Mechanism for JCV Transmigration across the Blood-Brain Barrier. J. Infect. Dis..

[11] White M.K., Khalili K. (2011). Pathogenesis of Progressive Multifocal Leukoencephalopathy—Revisited. J. Infect. Dis..

[12] Ferrante P., Caldarelli-Stefano R., Omodeo-Zorini E., Vago L., Boldorini R., Costanzi G. (1995). PCR Detection of JC Virus DNA in Brain Tissue from Patients with and without Progressive Multifocal Leukoencephalopathy. J. Med. Virol..

[13] Gorelik L., Reid C., Testa M., Brickelmaier M., Bossolasco S., Pazzi A., Bestetti A., Carmillo P., Wilson E., McAuliffe M. (2011). Progressive Multifocal Leukoencephalopathy (PML) Development Is Associated with Mutations in JC Virus Capsid Protein VP1 That Change Its Receptor Specificity. J. Infect. Dis..

[14] Khanna N., Elzi L., Mueller N.J., Garzoni C., Cavassini M., Fux C.A., Vernazza P., Bernasconi E., Battegay M., Hirsch H.H. (2009). Incidence and Outcome of Progressive Multifocal Leukoencephalopathy over 20 Years of the Swiss HIV Cohort Study. Clin. Infect. Dis..

[15] Anand P., Hotan G.C., Vogel A., Venna N., Mateen F.J. (2019). Progressive Multifocal Leukoencephalopathy: A 25-Year Retrospective Cohort Study. Neurol. Neuroimmunol. Neuroinflamm..

[16] Cortese I., Reich D.S., Nath A. (2021). Progressive Multifocal Leukoencephalopathy and the Spectrum of JC Virus-Related Disease. Nat. Rev. Neurol..

[17] Carson K.R., Evens A.M., Richey E.A., Habermann T.M., Focosi D., Seymour J.F., Laubach J., Bawn S.D., Gordon L.I., Winter J.N. (2009). Progressive Multifocal Leukoencephalopathy after Rituximab Therapy in HIV-Negative Patients: A Report of 57 Cases from the Research on Adverse Drug Events and Reports Project. Blood.

[18] Bloomgren G., Richman S., Hotermans C., Subramanyam M., Goelz S., Natarajan A., Lee S., Plavina T., Scanlon J.V., Sandrock A. (2012). Risk of Natalizumab-Associated Progressive Multifocal Leukoencephalopathy. N. Engl. J. Med..

[19] Pavlovic D., Patera A.C., Nyberg F., Gerber M., Liu M., Leukeoncephalopathy C.P.M. (2015). Progressive Multifocal Leukoencephalopathy: Current Treatment Options and Future Perspectives. Ther. Adv. Neurol. Diso..

[20] Tan I.L., Koralnik I.J., Rumbaugh J.A., Burger P.C., King-Rennie A., McArthur J.C. (2011). Progressive Multifocal Leukoencephalopathy in a Patient without Immunodeficiency. Neurology.

[21] Vermersch P., Kappos L., Gold R., Foley J.F., Olsson T., Cadavid D., Bozic C., Richman S. (2011). Clinical Outcomes of Natalizumab-Associated Progressive Multifocal Leukoencephalopathy(Podcast). Neurology.

[22] Prosperini L., de Rossi N., Scarpazza C., Moiola L., Cosottini M., Gerevini S., Capra R., Italian PML Study Group (2016). Study Natalizumab-Related Progressive Multifocal Leukoencephalopathy in Multiple Sclerosis: Findings from an Italian Independent Registry. PLoS ONE.

[23] Balduzzi A., Lucchini G., Hirsch H.H., Basso S., Cioni M., Rovelli A., Zincone A., Grimaldi M., Corti P., Bonanomi S. (2011). Polyomavirus JC-Targeted T-Cell Therapy for Progressive Multiple Leukoencephalopathy in a Hematopoietic Cell Transplantation Recipient. Bone Marrow Transpl..

[24] Muftuoglu M., Olson A., Marin D., Ahmed S., Mulanovich V., Tummala S., Chi T.L., Ferrajoli A., Kaur I., Li L. (2018). Allogeneic BK Virus–Specific T Cells for Progressive Multifocal Leukoencephalopathy. N. Engl. J. Med..

[25] Cortese I., Muranski P., Enose-Akahata Y., Ha S.-K., Smith B., Monaco M., Ryschkewitsch C., Major E.O., Ohayon J., Schindler M.K. (2019). Pembrolizumab Treatment for Progressive Multifocal Leukoencephalopathy. N. Engl. J. Med..

[26] Kondo Y., Windrem M.S., Zou L., Chandler-Militello D., Schanz S.J., Auvergne R.M., Betstadt S.J., Harrington A.R., Johnson M., Kazarov A. (2014). Human Glial Chimeric Mice Reveal Astrocytic Dependence of JC Virus Infection. J. Clin. Investig..

[27] Dyson N., Bernards R., Friend S.H., Gooding L.R., Hassell J.A., Major E.O., Pipas J.M., Vandyke T., Harlow E. (1990). Large T Antigens of Many Polyomaviruses Are Able to Form Complexes with the Retinoblastoma Protein. J. Virol..

[28] Valle L.D., Gordon J., Assimakopoulou M., Enam S., Geddes J.F., Varakis J.N., Katsetos C.D., Croul S., Khalili K. (2001). Detection of JC Virus DNA Sequences and Expression of the Viral Regulatory Protein T-Antigen in Tumors of the Central Nervous System. Cancer Res..

[29] Dickmanns A., Zeitvogel A., Simmersbach F., Weber R., Arthur A.K., Dehde S., Wildeman A.G., Fanning E. (1994). The Kinetics of Simian Virus 40-Induced Progression of Quiescent Cells into S Phase Depend on Four Independent Functions of Large T Antigen. J. Virol..

[30] Ferenczy M.W., Marshall L.J., Nelson C.D., Atwood W.J., Nath A., Khalili K., Major E.O. (2012). Molecular Biology, Epidemiology, and Pathogenesis of Progressive Multifocal Leukoencephalopathy, the JC Virus-Induced Demyelinating Disease of the Human Brain. Clin. Microbiol. Rev..

[31] Wilczek M.P., DuShane J.K., Armstrong F.J., Maginnis M.S. (2019). JC Polyomavirus Infection Reveals Delayed Progression of the Infectious Cycle in Normal Human Astrocytes. J. Virol..

[32] Lynch K.J., Frisque R.J. (1991). Factors Contributing to the Restricted DNA Replicating Activity of JC Virus. Virology.

[33] Sock E., Wegner M., Fortunato E.A., Grummt F. (1993). Large T-Antigen and Sequences within the Regulatory Region of JC Virus Both Contribute to the Features of JC Virus DNA Replication. Virology.

[34] Major E.O., Miller A.E., Mourrain P., Traub R.G., de Widt E., Sever J. (1985). Establishment of a Line of Human Fetal Glial Cells That Supports JC Virus Multiplication. Proc. Natl. Acad. Sci. USA.

[35] Ariza A., Mate J.L., Isamat M., Calatrava A., Fernandez-Vasalo A., Navas-Palacios J.J. (1998). Overexpression of Ki-67 and Cyclins A and B1 in JC Virus-Infected Cells of Progressive Multifocal Leukoencephalopathy. J. Neuropathol. Exp. Neurol..

[36] Sanchez V., McElroy A.K., Spector D.H. (2003). Mechanisms Governing Maintenance of Cdk1/Cyclin B1 Kinase Activity in Cells Infected with Human Cytomegalovirus. J. Virol..

[37] Marshall A., Rushbrook S., Davies S.E., Morris L.S., Scott I.S., Vowler S.L., Coleman N., Alexander G. (2005). Relation between Hepatocyte G1 Arrest, Impaired Hepatic Regeneration, and Fibrosis in Chronic Hepatitis C Virus Infection. Gastroenterology.

[38] Ahuja D., Sáenz-Robles M.T., Pipas J.M. (2005). SV40 Large T Antigen Targets Multiple Cellular Pathways to Elicit Cellular Transformation. Oncogene.

[39] Chang F., Lee J.T., Navolanic P.M., Steelman L.S., Shelton J.G., Blalock W.L., Franklin R.A., McCubrey J.A. (2003). Involvement of PI3K/Akt Pathway in Cell Cycle Progression, Apoptosis, and Neoplastic Transformation: A Target for Cancer Chemotherapy. Leukemia.

[40] Choudhury G.G., Karamitsos C., Hernandez J., Gentilini A., Bardgette J., Abboud H.E. (1997). PI-3-Kinase and MAPK Regulate Mesangial Cell Proliferation and Migration in Response to PDGF. Am. J. Physiol. Renal..

[41] Diehl J.A., Cheng M., Roussel M.F., Sherr C.J. (1998). Glycogen Synthase Kinase-3β Regulates Cyclin D1 Proteolysis and Subcellular Localization. Gene. Dev..

[42] Gille H., Downward J. (1999). Multiple Ras Effector Pathways Contribute to G1Cell Cycle Progression. J. Biol. Chem..

[43] Medema R.H., Kops G.J.P.L., Bos J.L., Burgering B.M.T. (2000). AFX-like Forkhead Transcription Factors Mediate Cell-Cycle Regulation by Ras and PKB through P27kip1. Nature.

[44] Muise-Helmericks R.C., Grimes H.L., Bellacosa A., Malstrom S.E., Tsichlis P.N., Rosen N. (1998). Cyclin D Expression Is Controlled Post-Transcriptionally via a Phosphatidylinositol 3-Kinase/Akt-Dependent Pathway*. J. Biol. Chem..

[45] Choudhury G.G. (2001). Akt Serine Threonine Kinase Regulates Platelet-Derived Growth Factor-Induced DNA Synthesis in Glomerular Mesangial Cells: Regulation of c-Fos and P27^Kip1^ Gene Expression. J. Biol. Chem..

[46] Yu Y., Alwine J.C. (2002). Human Cytomegalovirus Major Immediate-Early Proteins and Simian Virus 40 Large T Antigen Can Inhibit Apoptosis through Activation of the Phosphatidylinositide 3′-OH Kinase Pathway and the Cellular Kinase Akt. J. Virol..

[47] Yu Y., Alwine J.C. (2008). Interaction between Simian Virus 40 Large T Antigen and Insulin Receptor Substrate 1 Is Disrupted by the K1 Mutation, Resulting in the Loss of Large T Antigen-Mediated Phosphorylation of Akt. J. Virol..

[48] Clark P., Gee G.V., Albright B.S., Assetta B., Han Y., Atwood W.J., DiMaio D. (2020). Phosphoinositide 3′-Kinase γ Facilitates Polyomavirus Infection. Viruses.

[49] Vanhaesebroeck B., Guillermet-Guibert J., Graupera M., Bilanges B. (2010). The Emerging Mechanisms of Isoform-Specific PI3K Signalling. Nat. Rev. Mol. Cell Biol..

[50] Orellana J.A., Kwun H.J., Artusi S., Chang Y., Moore P.S. (2020). Sirolimus and Other MTOR Inhibitors Directly Activate Latent Pathogenic Human Polyomavirus Replication. J. Infect. Dis..

[51] Fruman D.A., Rommel C. (2014). PI3K and Cancer: Lessons, Challenges and Opportunities. Nat. Rev. Drug Discov..

[52] Yang J., Nie J., Ma X., Wei Y., Peng Y., Wei X. (2019). Targeting PI3K in Cancer: Mechanisms and Advances in Clinical Trials. Mol. Cancer.

[53] Oki Y., Fanale M., Romaguera J., Fayad L., Fowler N., Copeland A., Samaniego F., Kwak L.W., Neelapu S., Wang M. (2015). Phase II Study of an AKT Inhibitor MK2206 in Patients with Relapsed or Refractory Lymphoma. Brit. J. Haematol..

[54] Blagosklonny M.V. (2019). Rapamycin for Longevity: Opinion Article. Aging.

[55] Augustine J.J., Bodziak K.A., Hricik D.E. (2007). Use of Sirolimus in Solid Organ Transplantation. Drugs.

[56] Kastrati A., Mehilli J., von Beckerath N., Dibra A., Hausleiter J., Pache J., Schühlen H., Schmitt C., Dirschinger J., Schömig A. (2005). Sirolimus-Eluting Stent or Paclitaxel-Eluting Stent vs Balloon Angioplasty for Prevention of Recurrences in Patients with Coronary In-Stent Restenosis: A Randomized Controlled Trial. JAMA.

[57] Cloughesy T.F., Yoshimoto K., Nghiemphu P., Brown K., Dang J., Zhu S., Hsueh T., Chen Y., Wang W., Youngkin D. (2008). Antitumor Activity of Rapamycin in a Phase I Trial for Patients with Recurrent PTEN-Deficient Glioblastoma. PLoS Med..

[58] Kaeberlein M., Galvan V. (2019). Rapamycin and Alzheimer’s Disease: Time for a Clinical Trial?. Sci. Transl. Med..

[59] Peterson J.N., Lin B., Shin J., Phelan P.J., Tsichlis P., Schwob J.E., Bullock P.A. (2017). The Replication of JCV DNA in the G144 Oligodendrocyte Cell Line Is Dependent Upon Akt. J. Virol..

[60] Vacante D.A., Traub R., Major E.O. (1989). Extension of JC Virus Host Range to Monkey Cells by Insertion of a Simian Virus 40 Enhancer into the JC Virus Regulatory Region. Virology.

[61] DuShane J.K., Wilczek M.P., Mayberry C.L., Maginnis M.S. (2018). ERK Is a Critical Regulator of JC Polyomavirus Infection. J. Virol..

[62] Mayberry C.L., Wilczek M.P., Fong T.M., Nichols S.L., Maginnis M.S. (2021). GRK2 Mediates β-Arrestin Interactions with 5-HT 2 Receptors for JC Polyomavirus Endocytosis. J. Virol..

[63] Mayberry C.L., Soucy A.N., Lajoie C.R., DuShane J.K., Maginnis M.S. (2019). JC Polyomavirus Entry by Clathrin-Mediated Endocytosis Is Driven by β-Arrestin. J. Virol..

[64] DuShane J.K., Mayberry C.L., Wilczek M.P., Nichols S.L., Maginnis M.S. (2019). JCPyV-Induced MAPK Signaling Activates Transcription Factors during Infection. Int. J. Mol. Sci..

[65] DuShane J.K., Wilczek M.P., Crocker M.A., Maginnis M.S. (2019). High-Throughput Characterization of Viral and Cellular Protein Expression Patterns During JC Polyomavirus Infection. Front. Microbiol..

[66] Wilczek M.P., Armstrong F.J., Geohegan R.P., Mayberry C.L., DuShane J.K., King B.L., Maginnis M.S. (2021). The MAPK/ERK Pathway and the Role of DUSP1 in JCPyV Infection of Primary Astrocytes. Viruses.

[67] Robinson M.D., McCarthy D.J., Smyth G.K. (2010). EdgeR: A Bioconductor Package for Differential Expression Analysis of Digital Gene Expression Data. Bioinformatics.

[68] Kanehisa M., Furumichi M., Sato Y., Ishiguro-Watanabe M., Tanabe M. (2020). KEGG: Integrating Viruses and Cellular Organisms. Nucleic Acids Res..

[69] Kanehisa M., Goto S. (2000). KEGG: Kyoto Encyclopedia of Genes and Genomes. Nucleic Acids Res..

[70] Kanehisa M. (2019). Toward Understanding the Origin and Evolution of Cellular Organisms. Protein Sci..

[71] Howe K.L., Achuthan P., Allen J., Allen J., Alvarez-Jarreta J., Amode M.R., Armean I.M., Azov A.G., Bennett R., Bhai J. (2020). Ensembl 2021. Nucleic Acids Res..

[72] Larsson J. Eulerr: Area-Proportional Euler and Venn Diagrams with Ellipses. R Package Version 6.1.1, 2020. https://CRAN.R-project.org/package=eulerr.

[73] Shaul Y.D., Seger R. (2007). The MEK/ERK Cascade: From Signaling Specificity to Diverse Functions. Biochim. Biophys. Acta BBA Mol Cell Res..

[74] Querbes W., Benmerah A., Tosoni D., Fiore P.P.D., Atwood W.J. (2003). A JC Virus-Induced Signal Is Required for Infection of Glial Cells by a Clathrin- and Eps15-Dependent Pathway. J. Virol..

[75] McCubrey J.A., Lee J.T., Steelman L.S., Blalock W.L., Moye P.W., Chang F., Pearce M., Shelton J.G., White M.K., Franklin R.A. (2001). Interactions between the PI3K and Raf Signaling Pathways Can Result in the Transformation of Hematopoietic Cells. Cancer Detect Prev..

[76] Dangoria N.S., Breau W.C., Anderson H.A., Cishek D.M., Norkin L.C. (1996). Extracellular Simian Virus 40 Induces an ERK/MAP Kinase-Independent Signalling Pathway That Activates Primary Response Genes and Promotes Virus Entry. J. Gen. Virol..

[77] Rodriguez-Viciana P., Collins C., Fried M. (2006). Polyoma and SV40 Proteins Differentially Regulate PP2A to Activate Distinct Cellular Signaling Pathways Involved in Growth Control. Proc. Natl. Acad. Sci. USA.

[78] Mendoza M.C., Er E.E., Blenis J. (2011). The Ras-ERK and PI3K-MTOR Pathways: Cross-Talk and Compensation. Trends Biochem. Sci..

[79] Hayashi H., Tsuchiya Y., Nakayama K., Satoh T., Nishida E. (2008). Down-regulation of the PI3-kinase/Akt Pathway by ERK MAP Kinase in Growth Factor Signaling. Genes Cells.

[80] heon Rhim J., Luo X., Gao D., Xu X., Zhou T., Li F., Wang P., Wong S.T.C., Xia X. (2016). Cell Type-Dependent Erk-Akt Pathway Crosstalk Regulates the Proliferation of Fetal Neural Progenitor Cells. Sci. Rep.

[81] Turke A.B., Song Y., Costa C., Cook R., Arteaga C.L., Asara J.M., Engelman J.A. (2012). MEK Inhibition Leads to PI3K/AKT Activation by Relieving a Negative Feedback on ERBB Receptors. Cancer Res..

[82] Link A., Shin S.K., Nagasaka T., Balaguer F., Koi M., Jung B., Boland C.R., Goel A. (2009). JC Virus Mediates Invasion and Migration in Colorectal Metastasis. PLoS ONE.

[83] Martini M., De Santis M.C., Braccini L., Gulluni F., and Hirsch E. (2014). PI3K/AKT Signaling Pathway and Cancer: An Updated Review. Ann. Med..

[84] Foote M.B., White J.R., Jee J., Argilés G., Wan J.C.M., Rousseau B., Pessin M.S., Diaz L.A. (2021). Association of Antineoplasic Therapy with Decreased SARS-CoV-2 Infection Rates in Patients with Cancer. JAMA Oncol..

[85] Karam B.S., Morris R.S., Bramante C.T., Puskarich M., Zolfaghari E.J., Lotfi-Emran S., Ingraham N.E., Charles A., Odde D.J., Tignanelli C.J. (2021). MTOR Inhibition in COVID-19: A Commentary and Review of Efficacy in RNA Viruses. J. Med. Virol..

[86] Kindrachuk J., Ork B., Hart B.J., Mazur S., Holbrook M.R., Frieman M.B., Traynor D., Johnson R.F., Dyall J., Kuhn J.H. (2015). Antiviral Potential of ERK/MAPK and PI3K/AKT/MTOR Signaling Modulation for Middle East Respiratory Syndrome Coronavirus Infection as Identified by Temporal Kinome Analysis. Antimicrob. Agents Chemother..

[87] Appelberg S., Gupta S., Akusjärvi S.S., Ambikan A.T., Mikaeloff F., Saccon E., Végvári Á., Benfeitas R., Sperk M., Ståhlberg M. (2020). Dysregulation in Akt/MTOR/HIF-1 Signaling Identified by Proteo-Transcriptomics of SARS-CoV-2 Infected Cells. Emerg. Microbes Infec..

[88] Zhu N., Zhang D., Wang W., Li X., Yang B., Song J., Zhao X., Huang B., Shi W., Lu R. (2020). A Novel Coronavirus from Patients with Pneumonia in China, 2019. N. Engl. J Med..

[89] Liacini A., Seamone M.E., Muruve D.A., Tibbles L.A. (2010). Anti-BK Virus Mechanisms of Sirolimus and Leflunomide Alone and in Combination: Toward a New Therapy for BK Virus Infection. Transplantation.

[90] Andrabi S., Gjoerup O.V., Kean J.A., Roberts T.M., Schaffhausen B. (2007). Protein Phosphatase 2A Regulates Life and Death Decisions via Akt in a Context-Dependent Manner. Proc. Natl. Acad. Sci. USA.

[91] Bollag B., Hofstetter C.A., Reviriego-Mendoza M.M., Frisque R.J. (2010). JC Virus Small t Antigen Binds Phosphatase PP2A and Rb Family Proteins and Is Required for Efficient Viral DNA Replication Activity. PLoS ONE.

[92] Hirsch H.H., Yakhontova K., Lu M., Manzetti J. (2016). BK Polyomavirus Replication in Renal Tubular Epithelial Cells Is Inhibited by Sirolimus, but Activated by Tacrolimus Through a Pathway Involving FKBP-12. Am. J. Transplant..

[93] Hirsch H.H., Randhawa P., the AST Infectious Diseases Community of Practice (2013). BK Polyomavirus in Solid Organ Transplantation. Am. J. Transplant..

[94] Campistol J.M., Eris J., Oberbauer R., Friend P., Hutchison B., Morales J.M., Claesson K., Stallone G., Russ G., Rostaing L. (2006). Sirolimus Therapy after Early Cyclosporine Withdrawal Reduces the Risk for Cancer in Adult Renal Transplantation. J. Am. Soc. Nephrol..

[95] Zoncu R., Efeyan A., Sabatini D.M. (2011). MTOR: From Growth Signal Integration to Cancer, Diabetes and Ageing. Nat. Rev. Mol. Cell Biol..

[96] Pollard S.M., Yoshikawa K., Clarke I.D., Danovi D., Stricker S., Russell R., Bayani J., Head R., Lee M., Bernstein M. (2009). Glioma Stem Cell Lines Expanded in Adherent Culture Have Tumor-Specific Phenotypes and Are Suitable for Chemical and Genetic Screens. Cell Stem Cell.

[97] Louis N., Evelegh C., Graham F.L. (1997). Cloning and Sequencing of the Cellular–Viral Junctions from the Human Adenovirus Type 5 Transformed 293 Cell Line. Virology.

[98] Berk A.J. (2005). Recent Lessons in Gene Expression, Cell Cycle Control, and Cell Biology from Adenovirus. Oncogene.

[99] Moran E. (1993). DNA Tumor Virus Transforming Proteins and the Cell Cycle. Curr. Opin. Genet. Dev..

[100] Nevins J.R. (1992). E2F: A Link between the Rb Tumor Suppressor Protein and Viral Oncoproteins. Science.

[101] Frisch S.M., Mymryk J.S. (2002). Adenovirus-5 E1A: Paradox and Paradigm. Nat. Rev. Mol. Cell Biol..

[102] Levine A.J. (1990). The P53 Protein and Its Interactions with the Oncogene Products of the Small DNA Tumor Viruses. Virology.

[103] Arroyo J.D., Hahn W.C. (2005). Involvement of PP2A in Viral and Cellular Transformation. Oncogene.

[104] Sariyer I.K., Khalili K., Safak M. (2008). Dephosphorylation of JC Virus Agnoprotein by Protein Phosphatase 2A: Inhibition by Small t Antigen. Virology.

[105] Jhanwar-Uniya M., Wainwright J.V., Mohan A.L., Tobias M.E., Murali R., Gandhi C.D., Schmidt M.H. (2019). Diverse Signaling Mechanisms of MTOR Complexes: MTORC1 and MTORC2 in Forming a Formidable Relationship. Adv. Biol. Regul..

[106] Sarbassov D.D., Ali S.M., Kim D.-H., Guertin D.A., Latek R.R., Erdjument-Bromage H., Tempst P., Sabatini D.M. (2004). Rictor, a Novel Binding Partner of MTOR, Defines a Rapamycin-Insensitive and Raptor-Independent Pathway That Regulates the Cytoskeleton. Curr. Biol..

[107] Feldman M.E., Apsel B., Uotila A., Loewith R., Knight Z.A., Ruggero D., Shokat K.M. (2009). Active-Site Inhibitors of MTOR Target Rapamycin-Resistant Outputs of MTORC1 and MTORC2. PLoS Biol..

[108] Kwun H.J., Chang Y., Moore P.S. (2017). Protein-Mediated Viral Latency Is a Novel Mechanism for Merkel Cell Polyomavirus Persistence. Proc. Natl. Acad. Sci. USA.

[109] Wu J., Su H., Yu Z., Xi S., Guo C., Hu Z., Qu Y., Cai H., Zhao Y., Zhao H. (2020). Skp2 Modulates Proliferation, Senescence and Tumorigenesis of Glioma. Cancer Cell Int..

[110] Zhang L., Wang C. (2006). F-Box Protein Skp2: A Novel Transcriptional Target of E2F. Oncogene.

[111] Assetta B., Maginnis M.S., Ahufinger I.G., Haley S.A., Gee G.V., Nelson C.D.S., O’Hara B.A., Ramdial S.A.A., Atwood W.J. (2013). 5-HT2 Receptors Facilitate JC Polyomavirus Entry. J. Virol..

[112] Wipf P., Halter R.J. (2005). Chemistry and Biology of Wortmannin. Org. Biomol. Chem..

[113] Pittini Á., Casaravilla C., Allen J.E., Díaz Á. (2016). Pharmacological Inhibition of PI3K Class III Enhances the Production of Pro- and Anti-Inflammatory Cytokines in Dendritic Cells Stimulated by TLR Agonists. Int. Immunopharmacol..

[114] Brunn G.J., Williams J., Sabers C., Wiederrecht G., Lawrence J.C., Abraham R.T. (1996). Direct Inhibition of the Signaling Functions of the Mammalian Target of Rapamycin by the Phosphoinositide 3-kinase Inhibitors, Wortmannin and LY294002. EMBO J..

[115] O’Hara B.A., Morris-Love J., Gee G.V., Haley S.A., Atwood W.J. (2020). JC Virus Infected Choroid Plexus Epithelial Cells Produce Extracellular Vesicles That Infect Glial Cells Independently of the Virus Attachment Receptor. PLoS Pathog..

[116] Lei Y., Huang K., Gao C., Lau Q.C., Pan H., Xie K., Li J., Liu R., Zhang T., Xie N. (2011). Proteomics Identification of ITGB3 as a Key Regulator in Reactive Oxygen Species-Induced Migration and Invasion of Colorectal Cancer Cells. Mol. Cell Proteom..

[117] Fuentes P., Sesé M., Guijarro P.J., Emperador M., Sánchez-Redondo S., Peinado H., Hümmer S., y Cajal S.R. (2020). ITGB3-Mediated Uptake of Small Extracellular Vesicles Facilitates Intercellular Communication in Breast Cancer Cells. Nat. Commun..

[118] Van Niel G., D’Angelo G., Raposo G. (2018). Shedding Light on the Cell Biology of Extracellular Vesicles. Nat. Rev. Mol. Cell Biol..

[119] Mathieu M., Martin-Jaular L., Lavieu G., Théry C. (2019). Specificities of Secretion and Uptake of Exosomes and Other Extracellular Vesicles for Cell-to-Cell Communication. Nat. Cell. Biol..

[120] Zhang N., Ma D., Wang L., Zhu X., Pan Q., Zhao Y., Zhu W., Zhou J., Wang L., Chai Z. (2017). Insufficient Radiofrequency Ablation Treated Hepatocellular Carcinoma Cells Promote Metastasis by Up-Regulation ITGB3. J. Cancer.

[121] Anthony D.C., Ferguson B., Matyzak M.K., Miller K.M., Esiri M.M., Perry V.H. (1997). Differential Matrix Metalloproteinase Expression in Cases of Multiple Sclerosis and Stroke. Neuropath. Appl. Neurobiol..

[122] Anthony D.C., Miller K.M., Fearn S., Townsend M.J., Opdenakker G., Wells G.M.A., Clements J.M., Chandler S., Gearing A.J.H., Perry V.H. (1998). Matrix Metalloproteinase Expression in an Experimentally-Induced DTH Model of Multiple Sclerosis in the Rat CNS. J. Neuroimmunol..

[123] Maeda A., Sobel R.A. (1996). Matrix Metalloproteinases in the Normal Human Central Nervous System, Microglial Nodules, and Multiple Sclerosis Lesions. J. Neuropathol. Exp. Neurol..

